# *trans*-2,3-Bis(2,4,5-trimethyl-3-thien­yl)but-2-enedinitrile

**DOI:** 10.1107/S1600536811001176

**Published:** 2011-01-15

**Authors:** Jiang Bian, Ying Zhang, Xiaoyan Yan

**Affiliations:** a Department of Oral Biology, State University of New York at Buffalo, Buffalo, NY 14214, USA; b Department of Pharmaceutical Sciences, School of Pharmacy, State University of New York at Buffalo, Buffalo, NY 14260, USA; c Department of Biology, Taiyuan Normal University, Taiyuan 030012, People’s Republic of China

## Abstract

In title compound, C_18_H_18_N_2_S_2_, the dihedral angle between two thio­phene rings is 61.83 (8)°.

## Related literature

For related structures, see: Munakata *et al.* (1996 ▶); Han *et al.* (2006 ▶).
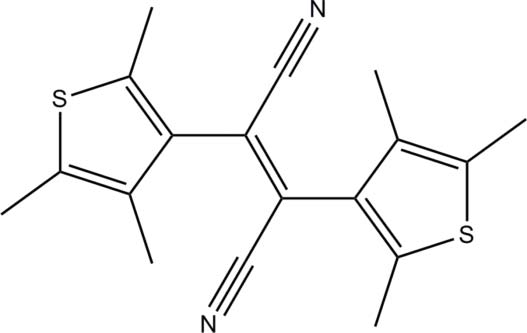


## Experimental

### 

#### Crystal data

C_18_H_18_N_2_S_2_
*M* *_r_* = 326.46
Triclinic, 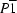

*a* = 8.8368 (10) Å
*b* = 9.1785 (10) Å
*c* = 11.4160 (12) Å
α = 85.271 (2)°
β = 71.058 (2)°
γ = 77.171 (2)°
*V* = 853.88 (16) Å^3^
*Z* = 2
Mo *K*α radiation
μ = 0.31 mm^−1^
*T* = 273 K
0.40 × 0.32 × 0.28 mm

#### Data collection

Bruker SMART CCD area-detector diffractometer
Absorption correction: multi-scan (*SADABS*; Sheldrick, 1996 ▶) *T*_min_ = 0.886, *T*_max_ = 0.918
5025 measured reflections
3686 independent reflections
2264 reflections with *I* > 2σ(*I*)
*R*_int_ = 0.020

#### Refinement

*R*[*F* ^2^ > 2σ(*F* ^2^)] = 0.058
*wR*(*F* ^2^) = 0.175
*S* = 1.06
3686 reflections
199 parameters
H-atom parameters constrained
Δρ_max_ = 0.28 e Å^−3^
Δρ_min_ = −0.23 e Å^−3^

### 

Data collection: *SMART* (Bruker, 1998 ▶); cell refinement: *SAINT* (Bruker, 1999 ▶); data reduction: *SAINT*; program(s) used to solve structure: *SHELXTL* (Sheldrick, 2008 ▶); program(s) used to refine structure: *SHELXTL*; molecular graphics: *SHELXTL*; software used to prepare material for publication: *SHELXTL*.

## Supplementary Material

Crystal structure: contains datablocks global, I. DOI: 10.1107/S1600536811001176/hg2788sup1.cif

Structure factors: contains datablocks I. DOI: 10.1107/S1600536811001176/hg2788Isup2.hkl

Additional supplementary materials:  crystallographic information; 3D view; checkCIF report

## Figures and Tables

**Fig. 1.**
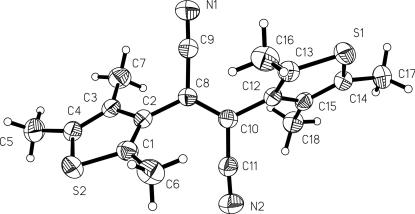
View of the title compound (I), with displacement ellipsoids drawn at the 40% probability level.

## supplementary crystallographic information

### Comment

In the crystal structure of the title compound, two substituted thiophene rings are *trans* positioned with respect to the dicyano group. The ring skeleton of the molecule is not planar. This diarylethene with thiophene rings is prepared in an attempt to construct thermally irreversible photochromic systems. The dicyano group was selected to shift the absorption maxima of the dihydro-type isomers to longer wavelengths.

All bond lengths and angles in title compound are normal and good agreement with those previously reported. (Munakata, *et al.*, 1996; Han *et al.*, 2006). The dihedral angles between the two thiophene (S1/C12—C15 and S2/C1—C4) rings is 61.83 °. No classical hydrogen bonds were found, the crystal structure was mainly stabilized by Van der Waals forces.

### Experimental

To 20 ml of 50% NaOH aqueous solution containing triethylbenzylammonium chloride (0.21 g, 0.0010 mol) was added a mixture of 2,3,5-trimethyl-4-(cyanomethyl)thiophene (16 g, 0.10 mol) and CCl4 (15 g, 0.10 mol) at 40 ° C. The solution was stirred for 1.5 h at 45 ° C. The reaction mixture was poured into water and the product was extracted with ether and chloroform. After the solvent was removed, the mixture of *trans* and *cis* forms was separated by column chlomatography on silica gel with light petroleum-CHCl3 (1: 1), collected the first yellow band, and then purified by recrystallization from a hexane-ether mixture. Single crystals suitable for X-ray measurements were obtained by recrystallization from methanol at room temperature for one week.

### Refinement

H atoms bonded to C atoms were treated as riding atoms, with C—H distances of 0.96 Å and *U*_iso_(H) values of 1.5*U*_eq_(C).

### Crystal data

**Table:**

C_18_H_18_N_2_S_2_	*Z* = 2
*M**_r_* = 326.46	*F*(000) = 344
Triclinic, *P*1	*D*_x_ = 1.270 Mg m^−^^3^
Hall symbol: -P 1	Mo *K*α radiation, λ = 0.71073 Å
*a* = 8.8368 (10) Å	Cell parameters from 2501 reflections
*b* = 9.1785 (10) Å	θ = 2.3–25.1°
*c* = 11.4160 (12) Å	µ = 0.31 mm^−^^1^
α = 85.271 (2)°	*T* = 273 K
β = 71.058 (2)°	Block, yellow
γ = 77.171 (2)°	0.40 × 0.32 × 0.28 mm
*V* = 853.88 (16) Å^3^	

### Data collection

**Table:**

Bruker SMART CCD area-detector diffractometer	3686 independent reflections
Radiation source: fine-focus sealed tube	2264 reflections with *I* > 2σ(*I*)
graphite	*R*_int_ = 0.020
φ and ω scans	θ_max_ = 27.5°, θ_min_ = 1.9°
Absorption correction: multi-scan (*SADABS*; Sheldrick, 1996)	*h* = −11→9
*T*_min_ = 0.886, *T*_max_ = 0.918	*k* = −11→11
5025 measured reflections	*l* = −13→14

### Refinement

**Table:**

Refinement on *F*^2^	Primary atom site location: structure-invariant direct methods
Least-squares matrix: full	Secondary atom site location: difference Fourier map
*R*[*F*^2^ > 2σ(*F*^2^)] = 0.058	Hydrogen site location: inferred from neighbouring sites
*wR*(*F*^2^) = 0.175	H-atom parameters constrained
*S* = 1.06	*w* = 1/[σ^2^(*F*_o_^2^) + (0.0826*P*)^2^ + 0.1249*P*] where *P* = (*F*_o_^2^ + 2*F*_c_^2^)/3
3686 reflections	(Δ/σ)_max_ < 0.001
199 parameters	Δρ_max_ = 0.28 e Å^−^^3^
0 restraints	Δρ_min_ = −0.23 e Å^−^^3^

### Special details

**Table:**

Geometry. All e.s.d.'s (except the e.s.d. in the dihedral angle between two l.s. planes) are estimated using the full covariance matrix. The cell e.s.d.'s are taken into account individually in the estimation of e.s.d.'s in distances, angles and torsion angles; correlations between e.s.d.'s in cell parameters are only used when they are defined by crystal symmetry. An approximate (isotropic) treatment of cell e.s.d.'s is used for estimating e.s.d.'s involving l.s. planes.
Refinement. Refinement of *F*^2^ against ALL reflections. The weighted *R*-factor *wR* and goodness of fit *S* are based on *F*^2^, conventional *R*-factors *R* are based on *F*, with *F* set to zero for negative *F*^2^. The threshold expression of *F*^2^ > σ(*F*^2^) is used only for calculating *R*-factors(gt) *etc*. and is not relevant to the choice of reflections for refinement. *R*-factors based on *F*^2^ are statistically about twice as large as those based on *F*, and *R*- factors based on ALL data will be even larger.

### Fractional atomic coordinates and isotropic or equivalent isotropic displacement parameters (Å^2^)

**Table:**

	*x*	*y*	*z*	*U*_iso_*/*U*_eq_	
S1	0.36871 (11)	0.71963 (9)	0.60411 (8)	0.0553 (3)	
S2	0.40282 (12)	−0.15478 (10)	0.92480 (9)	0.0616 (3)	
C12	0.2777 (4)	0.4790 (3)	0.6886 (3)	0.0409 (7)	
C2	0.2998 (4)	0.1229 (3)	0.8994 (3)	0.0428 (7)	
C10	0.2760 (4)	0.3248 (3)	0.7386 (3)	0.0420 (7)	
C15	0.1480 (4)	0.5656 (3)	0.6474 (2)	0.0410 (7)	
C13	0.4056 (4)	0.5472 (4)	0.6719 (3)	0.0474 (8)	
C8	0.2843 (4)	0.2788 (3)	0.8535 (3)	0.0438 (7)	
C11	0.2615 (4)	0.2192 (4)	0.6591 (3)	0.0481 (8)	
C14	0.1819 (4)	0.6995 (3)	0.5987 (3)	0.0459 (7)	
C3	0.1900 (4)	0.0800 (3)	1.0143 (3)	0.0457 (7)	
C1	0.4219 (4)	0.0072 (3)	0.8411 (3)	0.0481 (8)	
C4	0.2314 (4)	−0.0692 (4)	1.0389 (3)	0.0527 (8)	
C18	−0.0090 (4)	0.5183 (4)	0.6644 (3)	0.0556 (9)	
H18A	−0.0777	0.5931	0.6298	0.083*	
H18B	−0.0636	0.5061	0.7512	0.083*	
H18C	0.0133	0.4252	0.6232	0.083*	
N2	0.2477 (4)	0.1424 (3)	0.5914 (3)	0.0682 (9)	
C9	0.2797 (5)	0.3908 (4)	0.9369 (3)	0.0543 (8)	
C7	0.0454 (5)	0.1843 (4)	1.0961 (3)	0.0654 (10)	
H7A	−0.0088	0.1304	1.1673	0.098*	
H7B	−0.0292	0.2258	1.0509	0.098*	
H7C	0.0816	0.2635	1.1223	0.098*	
N1	0.2708 (5)	0.4759 (4)	1.0067 (3)	0.0814 (11)	
C6	0.5626 (4)	0.0063 (4)	0.7253 (3)	0.0651 (10)	
H6A	0.5563	0.1048	0.6892	0.098*	
H6B	0.5588	−0.0623	0.6677	0.098*	
H6C	0.6632	−0.0242	0.7444	0.098*	
C17	0.0801 (5)	0.8252 (4)	0.5472 (3)	0.0655 (10)	
H17A	−0.0200	0.7979	0.5506	0.098*	
H17B	0.1395	0.8446	0.4628	0.098*	
H17C	0.0558	0.9133	0.5953	0.098*	
C16	0.5635 (4)	0.4920 (4)	0.6983 (4)	0.0648 (10)	
H16A	0.5649	0.3945	0.7361	0.097*	
H16B	0.5745	0.5595	0.7536	0.097*	
H16C	0.6526	0.4866	0.6223	0.097*	
C5	0.1468 (6)	−0.1591 (4)	1.1464 (3)	0.0789 (12)	
H5A	0.0541	−0.0947	1.2009	0.118*	
H5B	0.2214	−0.2043	1.1906	0.118*	
H5C	0.1108	−0.2356	1.1164	0.118*	

### Atomic displacement parameters (Å^2^)

**Table:**

	*U*^11^	*U*^22^	*U*^33^	*U*^12^	*U*^13^	*U*^23^
S1	0.0595 (6)	0.0513 (5)	0.0576 (5)	−0.0242 (4)	−0.0145 (4)	0.0040 (4)
S2	0.0718 (6)	0.0429 (5)	0.0699 (6)	−0.0036 (4)	−0.0276 (5)	−0.0013 (4)
C12	0.0455 (17)	0.0419 (17)	0.0338 (15)	−0.0106 (14)	−0.0097 (13)	−0.0001 (12)
C2	0.0490 (18)	0.0417 (17)	0.0398 (16)	−0.0079 (14)	−0.0178 (14)	−0.0006 (13)
C10	0.0425 (17)	0.0417 (16)	0.0412 (16)	−0.0105 (13)	−0.0110 (13)	−0.0025 (13)
C15	0.0431 (17)	0.0462 (17)	0.0327 (15)	−0.0112 (14)	−0.0094 (13)	−0.0006 (13)
C13	0.0443 (18)	0.0493 (19)	0.0475 (17)	−0.0128 (15)	−0.0104 (14)	−0.0026 (14)
C8	0.0510 (18)	0.0394 (16)	0.0399 (16)	−0.0092 (14)	−0.0126 (14)	−0.0028 (13)
C11	0.055 (2)	0.0485 (19)	0.0433 (17)	−0.0162 (15)	−0.0156 (15)	0.0022 (15)
C14	0.0520 (19)	0.0447 (18)	0.0411 (16)	−0.0101 (15)	−0.0151 (14)	0.0010 (14)
C3	0.0533 (19)	0.0490 (18)	0.0367 (16)	−0.0127 (15)	−0.0153 (14)	−0.0003 (13)
C1	0.0486 (19)	0.0449 (18)	0.0508 (18)	−0.0058 (15)	−0.0169 (15)	−0.0058 (15)
C4	0.069 (2)	0.0485 (19)	0.0479 (18)	−0.0186 (17)	−0.0261 (16)	0.0059 (15)
C18	0.051 (2)	0.063 (2)	0.058 (2)	−0.0173 (17)	−0.0203 (16)	0.0035 (17)
N2	0.084 (2)	0.066 (2)	0.0641 (19)	−0.0231 (17)	−0.0289 (17)	−0.0100 (17)
C9	0.073 (2)	0.0437 (18)	0.0458 (18)	−0.0077 (17)	−0.0213 (17)	−0.0013 (15)
C7	0.072 (2)	0.065 (2)	0.051 (2)	−0.0134 (19)	−0.0064 (18)	−0.0075 (17)
N1	0.130 (3)	0.057 (2)	0.060 (2)	−0.019 (2)	−0.032 (2)	−0.0110 (17)
C6	0.050 (2)	0.064 (2)	0.070 (2)	−0.0069 (18)	−0.0050 (18)	−0.0098 (19)
C17	0.084 (3)	0.051 (2)	0.063 (2)	−0.0120 (19)	−0.029 (2)	0.0108 (17)
C16	0.050 (2)	0.069 (2)	0.081 (3)	−0.0162 (18)	−0.0243 (19)	−0.006 (2)
C5	0.117 (4)	0.067 (3)	0.060 (2)	−0.038 (2)	−0.031 (2)	0.017 (2)

### Geometric parameters (Å, °)

**Table:**

S1—C13	1.716 (3)	C4—C5	1.503 (5)
S1—C14	1.723 (3)	C18—H18A	0.9600
S2—C1	1.711 (3)	C18—H18B	0.9600
S2—C4	1.723 (4)	C18—H18C	0.9600
C12—C13	1.364 (4)	C9—N1	1.133 (4)
C12—C15	1.432 (4)	C7—H7A	0.9600
C12—C10	1.483 (4)	C7—H7B	0.9600
C2—C1	1.370 (4)	C7—H7C	0.9600
C2—C3	1.441 (4)	C6—H6A	0.9600
C2—C8	1.476 (4)	C6—H6B	0.9600
C10—C8	1.364 (4)	C6—H6C	0.9600
C10—C11	1.433 (4)	C17—H17A	0.9600
C15—C14	1.361 (4)	C17—H17B	0.9600
C15—C18	1.493 (4)	C17—H17C	0.9600
C13—C16	1.492 (5)	C16—H16A	0.9600
C8—C9	1.443 (4)	C16—H16B	0.9600
C11—N2	1.140 (4)	C16—H16C	0.9600
C14—C17	1.502 (4)	C5—H5A	0.9600
C3—C4	1.367 (4)	C5—H5B	0.9600
C3—C7	1.500 (5)	C5—H5C	0.9600
C1—C6	1.491 (5)		
			
C13—S1—C14	92.95 (15)	H18A—C18—H18B	109.5
C1—S2—C4	93.19 (15)	C15—C18—H18C	109.5
C13—C12—C15	114.4 (3)	H18A—C18—H18C	109.5
C13—C12—C10	123.2 (3)	H18B—C18—H18C	109.5
C15—C12—C10	122.3 (3)	N1—C9—C8	176.8 (4)
C1—C2—C3	113.7 (3)	C3—C7—H7A	109.5
C1—C2—C8	124.0 (3)	C3—C7—H7B	109.5
C3—C2—C8	122.3 (3)	H7A—C7—H7B	109.5
C8—C10—C11	118.9 (3)	C3—C7—H7C	109.5
C8—C10—C12	124.9 (3)	H7A—C7—H7C	109.5
C11—C10—C12	116.2 (2)	H7B—C7—H7C	109.5
C14—C15—C12	111.4 (3)	C1—C6—H6A	109.5
C14—C15—C18	124.7 (3)	C1—C6—H6B	109.5
C12—C15—C18	123.8 (3)	H6A—C6—H6B	109.5
C12—C13—C16	130.3 (3)	C1—C6—H6C	109.5
C12—C13—S1	109.8 (2)	H6A—C6—H6C	109.5
C16—C13—S1	119.8 (2)	H6B—C6—H6C	109.5
C10—C8—C9	117.8 (3)	C14—C17—H17A	109.5
C10—C8—C2	125.3 (3)	C14—C17—H17B	109.5
C9—C8—C2	116.9 (3)	H17A—C17—H17B	109.5
N2—C11—C10	175.8 (3)	C14—C17—H17C	109.5
C15—C14—C17	129.5 (3)	H17A—C17—H17C	109.5
C15—C14—S1	111.4 (2)	H17B—C17—H17C	109.5
C17—C14—S1	119.1 (3)	C13—C16—H16A	109.5
C4—C3—C2	111.6 (3)	C13—C16—H16B	109.5
C4—C3—C7	123.7 (3)	H16A—C16—H16B	109.5
C2—C3—C7	124.6 (3)	C13—C16—H16C	109.5
C2—C1—C6	130.3 (3)	H16A—C16—H16C	109.5
C2—C1—S2	110.3 (2)	H16B—C16—H16C	109.5
C6—C1—S2	119.4 (2)	C4—C5—H5A	109.5
C3—C4—C5	128.6 (3)	C4—C5—H5B	109.5
C3—C4—S2	111.2 (2)	H5A—C5—H5B	109.5
C5—C4—S2	120.2 (3)	C4—C5—H5C	109.5
C15—C18—H18A	109.5	H5A—C5—H5C	109.5
C15—C18—H18B	109.5	H5B—C5—H5C	109.5
			
C13—C12—C10—C8	61.6 (4)	C12—C15—C14—C17	−178.7 (3)
C15—C12—C10—C8	−121.9 (3)	C18—C15—C14—C17	−2.8 (5)
C13—C12—C10—C11	−119.5 (3)	C12—C15—C14—S1	−0.3 (3)
C15—C12—C10—C11	57.0 (4)	C18—C15—C14—S1	175.6 (2)
C13—C12—C15—C14	0.4 (4)	C13—S1—C14—C15	0.1 (2)
C10—C12—C15—C14	−176.3 (3)	C13—S1—C14—C17	178.7 (3)
C13—C12—C15—C18	−175.5 (3)	C1—C2—C3—C4	−0.9 (4)
C10—C12—C15—C18	7.7 (4)	C8—C2—C3—C4	−178.3 (3)
C15—C12—C13—C16	−177.6 (3)	C1—C2—C3—C7	−179.5 (3)
C10—C12—C13—C16	−0.9 (5)	C8—C2—C3—C7	3.1 (5)
C15—C12—C13—S1	−0.3 (3)	C3—C2—C1—C6	−176.8 (3)
C10—C12—C13—S1	176.4 (2)	C8—C2—C1—C6	0.6 (5)
C14—S1—C13—C12	0.1 (2)	C3—C2—C1—S2	0.6 (3)
C14—S1—C13—C16	177.7 (3)	C8—C2—C1—S2	177.9 (2)
C11—C10—C8—C9	−173.0 (3)	C4—S2—C1—C2	−0.1 (3)
C12—C10—C8—C9	5.9 (5)	C4—S2—C1—C6	177.6 (3)
C11—C10—C8—C2	7.8 (5)	C2—C3—C4—C5	−178.2 (3)
C12—C10—C8—C2	−173.3 (3)	C7—C3—C4—C5	0.4 (5)
C1—C2—C8—C10	55.5 (5)	C2—C3—C4—S2	0.8 (3)
C3—C2—C8—C10	−127.3 (3)	C7—C3—C4—S2	179.4 (3)
C1—C2—C8—C9	−123.7 (3)	C1—S2—C4—C3	−0.4 (3)
C3—C2—C8—C9	53.5 (4)	C1—S2—C4—C5	178.7 (3)
C8—C10—C11—N2	167 (5)	C10—C8—C9—N1	133 (7)
C12—C10—C11—N2	−12 (5)	C2—C8—C9—N1	−48 (7)
